# Evaluation of methylenetetrahydrofolate reductase and S-adenosyl-methionine level in male infertility: A case-control study

**DOI:** 10.18502/ijrm.v20i4.10902

**Published:** 2022-05-23

**Authors:** Khadijeh Baranizadeh, Maryam Bahmanzadeh, Heidar Tavilani, Tayebeh Ghiasvand, Iraj Amiri, Mahnaz Yavangi, Gholamreza Shafiee

**Affiliations:** ^1^Department of Clinical Biochemistry, Medicine School, Hamadan University of Medical Sciences, Hamadan, Iran.; ^2^Department of Anatomical Sciences, School of Medicine, Endometrium and Endometriosis Research Center, Hamadan University of Medical Sciences, Hamadan, Iran.; ^3^Department of Obstetrics and Gynecology, School of Medicine, Endometrium and Endometriosis Research Center, Fatemieh Hospital, Hamadan University of Medical Sciences, Hamadan, Iran.; ^4^Department of Clinical Biochemistry, Medicine School, Nutrition Health Research Center, Hamadan University of Medical Sciences, Hamadan, Iran.

**Keywords:** Methylenetetrahydrofolate reductase, S-adenosylmethionine, Normozoospermia, Oligozoospermia, Folic acid.

## Abstract

**Background:**

Methylenetetrahydrofolate reductase enzyme (MTHFR) plays a key role in regulating folate balance, converting homocysteine to methionine, and producing s-adenosylmethionine (SAM) that plays a role in the methylation process.

**Objective:**

This study aimed to determine MTHFR activity and SAM level in men with normozoospermia and oligozoospermia.

**Materials and Methods:**

30 oligozoospermic and 30 normozoospermic men as controls were enrolled in this case-control study. Semen analysis was conducted according to the world health organization criteria. All semen samples were collected after 3-5 days of sexual abstinence. The sperms were evaluated by sperm test video software. All subjects SAM level was measured by enzyme-linked immunosorbent assay kit, and MTHFR were measured manually.

**Results:**

2 groups had a significant difference in sperm morphology (p = 0.02), concentration (p = 0.02) and motility (p = 0.03). The MTHFR activity in normozoospermic and oligozoospermic groups had significantly differences (p = 0.01). The level of SAM in the semen of oligozoospermic men was statistically lower than normozoospermic men (p = 0.03). Also, there was a positive association between MTHFR enzyme activity and SAM level in the normozoospermia group (p = 0.02, β = 0.67) and oligozoospermia group (p = 0.03, β = 0.54).

**Conclusion:**

MTHFR activity and SAM concentration were statistically lower in oligozoospermia men. It seems they can affect sperm concentration, morphology, and motility.

## 1. Introduction

Infertility is the inability of a couple to get pregnant after one year of unprotected intercourse without contraceptive methods and despite adequate intercourse (1). Infertility is a disorder that affects about 30-50% of men in cases overall (2).

Defects in spermatogenesis are one of the causes of infertility, and folate is important in this process (3). Folate and B12 play the main role in the methylation of uracil to the production of thymine in the DNA structure (4). Methylenetetrahydrofolate reductase (MTHFR) is a key enzyme in the biochemical pathway of one-carbon metabolism (5) and the storage of methyl groups for DNA methylation (6).

DNA methylation is one of the important factors in regulating gene expression (7). This enzyme has enzyme committee number 1, 1, 99, 15, and catalyzes the reduction of 5, 10 MTHF to 5-methyltetrahydrofolate using nicotinamide adenine dinucleotide phosphate (NADPH), which is an irreversible reaction. The methyl group of 5-methyl tetrahydrofolate is transferred to homocysteine for producing methionine, and subsequently, methionine is used to form s-adenosine methionine (SAM) (8).

SAM acts as a methyl group donor for DNA methylation (9, 10). SAM is a methyl group source for thymidylate biosynthesis and SAM-dependent methylation (11). The methylation process is very important for the regulation of DNA transcription, histone modification, and stabilization of the genome, so it is tightly regulated (12). It seems that mutation or decreased activity of the MTHFR enzyme leads to a decrease in S-adenosylmethionine and DNA methylation, ultimately disrupting the spermatogenesis pathway (13).

Therefore, we evaluated MTHFR activity and the S-adenosylmethionine level in normozoospermic and oligozoospermic men.

## 2. Material and Methods

### Collection of samples

This observational study recruited, semen samples of normozoospermic (n = 30) and oligozoospermic men (n = 30) from the endometrium and endometriosis center, Hamadan, Iran between May 2019 and August 2021. All subjects were evaluated using a questionnaire covering fertility parameters, medical history, and chronic diseases.

Participants with recognizable causes of male infertility such as obstructive oligozoospermia, varicocele, infections, and diabetes were excluded. Normozoospermic men were defined as samples with motility > 40%, morphology > 4%, and sperm concentration > 15 million/ml were included as normozoospermia, and samples low of these parameters were selected as oligozoospermia. Semen analysis was conducted according to the 2010 World Health Organization criteria (14). All semen samples were collected in sterile containers after 3-5 days of sexual abstinence. The samples were then incubated in a 37 C incubator for 30-40 min. Subsequently, semen liquid macroscopic tests were initially performed (15).

The number of samples required for this study was calculated based on the dependent variable of plasma S-adenosylmethionine concentration. The sample size was calculated based on the deviation of the criteria obtained from previous studies and using the following formula (16, 17):


$$n=\frac{{\left(Z_{1-\alpha /2}+Z_{1-\beta }\right)}^{2}{\left(\sigma_{1}^{2}+\sigma_{2}^{2}\right)}^{}}{{\left(\mu_{1}-\mu_{2}\right)}^{2}},\alpha =5\%,Z_{1-\alpha /2}=1.96,\beta =10\%,Z_{1-\beta }=1.23$$

### Sperm parameters 

For grouping the individuals, semen fluid analysis was performed, and the parameters of sperm count, motility, and morphology were evaluated based on that, individuals were divided into 2 groups: normozoospermic and oligozoospermic.

### Sperm count and motility 

The concentration and motility of spermatozoa were evaluated using a computer-assisted sperm analysis system by sperm test video software. At first, a 3 μl sample was loaded into a 20 μm slide at 37 C for analyses at 30 min intervals up to 180 min. Manual sampling was also performed to ensure the accuracy of the semen analyzer (18).

### Sperm morphology 

The sperm morphology was evaluated by quick-diff dye solutions. At first, a drop of the above samples was smeared then drying, and staining was performed. The dried slides were incubated in fixation solution for 75 sec, then in staining solution for 60 sec, and finally in detaining solution for 35 sec. After washing with distilled water and drying, their appearance was shown by microscope (15).

### Preparation of seminal plasma

First, semen samples were centrifuged at 500 g for 10 min to separate the semen plasma; simultaneously were taken and were maintained at -20 C (19).

### Seminal plasma measurement of MTHFR enzymatic activity

For detection of MTHFR enzymatic activity, seminal plasma was measured by a previous method with modifications (20). First, the 96 well plates were filled with 30 μL formaldehyde, 100 μL Tetrahydrofolate, and 200 μL phosphate buffer saline. The plate was incubated at 37 C for 5 min, and 200 μL flavine adenine dinucleotide and 200 μL ascorbic acid with 100 μL 2-mercaptoethanol were added to all wells, then 6 μL samples were added except the blank tube. The tubes were placed at 37 C for 5 min. Finally, 20 μL NADPH was added except the blanks, re-incubated at 37 C for 5 min and immediately the absorbance of the samples was measured at 340 nm by an enzyme-linked immunosorbent assay (ELISA) reader (Tecan Group Ltd, Männedorf-Switzerland).

### Measurement of SAM

Semen SAM level was measured using sigma SAM ELISA kit. First, was added 40 µl of the semen sample and 10 µl of SAM-Antibody and 50 µl of streptavidin to the test well and was added 50 µl standard and 50 µl streptavidin to standard well. After the incubation of well sat 37 C for 60 min and washing 30 sec, 50 µl of chromogen A and 50 µl of chromogen B were added to each well and incubated at 37 C for 10 min. Finally, add 50 ml of stop solution was added to each well and measured absorbance at 450 nm by a Sunrise ${}^{TM}$ ELISA plate reader (Tecan Group Ltd, Männedorf-Switzerland).

### Ethical considerations

Written informed consent was obtained from each participants. Approval was obtained from the Ethics Committee of Hamadan University of Medical Sciences, Hamadan, Iran (Code: IR.UMSHA.REC.1396.361).

### Statistical analysis

Data were analyzed using statistical software by Statistical Package for the Social Sciences version 16.0 (SPSS Inc., Chicago, USA), and Shapiro-Wilks tests were used to determine the data normality. Sample ${}^{{}^{'}}$ s *t* test was used to analyze the data and compare 2 groups, and association tests were used to investigate the relationship between the variables. Results were presented as mean ± SD, and the p < 0.05 was considered significant.

## 3. Results

### Demographic data

In the present study, it was found that the data were normal after Shapiro-Wilks tests analysis. The minimum age of men in the 2 groups was 32 yr, and a maximum of 42 yr and the mean age of the men was 37 yr, and the 2 study groups were matched by age (p = 0.15). The results of semen analysis are shown in table I. The 2 groups had a significant difference in sperm morphology (p = 0.02), concentration (p = 0.02) and motility (p = 0.03). Although the semen volume and pH were no significant difference in the 2 groups.

### Semen MTHFR enzyme activity

**Figure 1:**
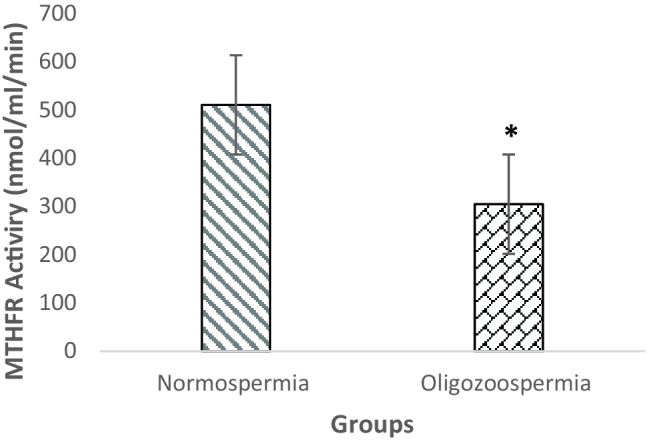
Comparison of MTHFR enzyme activity between groups. *P < 0.05.

Our results showed that MTHFR activity in the normozoospermia group was 510.66 ± 43.86 nmol/ml/min, while in oligozoospermia, men were 304.9 ± 29.75 nmol/ml/min. The mean of MTHFR activity was significantly different between men with normozoospermia and oligozoospermia groups (p = 0.01) (Figure 1).

### Semen SAM levels

**Figure 2:**
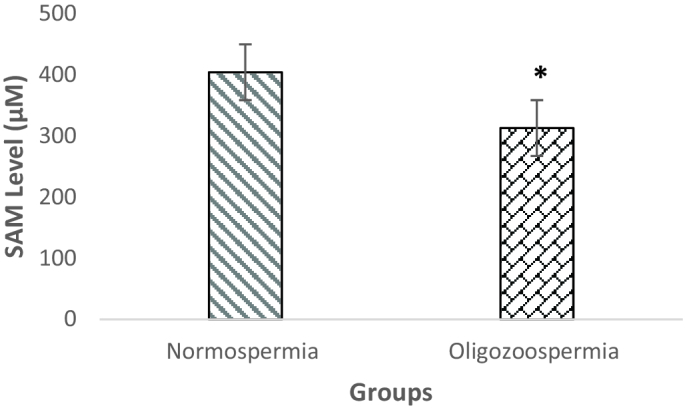
Comparison of serum S-adenosine methionine levels between groups. *P < 0.05.

SAM level in the seminal plasma of oligozoospermia was 312.65 ± 80.27 µmol/l ${}^{-1}$ and in normozoospermia men was 403.84 ± 86 µmol/l ${}^{-1}$ . SAM level was significantly lower in oligozoospermia men compared with normozoospermia men (p = 0.03) (Figure 2). According to association analysis, there was a positive association between MTHFR enzyme activity and SAM level in the normozoospermia group (p = 0.02, β = 0.67). Also, statistical analysis showed that in the oligozoospermia group, similar to normozoospermia men, there was a positive association between MTHFR enzyme activity and SAM level (p = 0.03, β = 0.54).

**Table 1:** Spermogram parameters in 2 groups of normozoospermia and oligozoospermia men


**Variable**	**Normozoospermia group **	**Oligozoospermia group **	**P-value**
**Age (yr)**	37.04 ± 4.99	37.14 ± 4.89	0.15
**Semen pH**	7.5 ± 0.13	7.5 ± 0.34	0.17
**Sperm morphology (%)**	37.5 ± 4.41	13.5 ± 3.23	0.02*
**Semen volume (ml)**	3.67 ± 0.87	4.09 ± 1.23	0.10
**Sperm motility % **	35.7 ± 4.3	27.3 ± 2.7	0.03*
**Sperm concentration (million/ml)**	57.39 ± 6.58	14.86 ± 3.58	0.02*
Data presented as Mean ± SD. Sample's *t* test, *P < 0.05			

## 4. Discussion

The results of spermogram parameters showed that the mean of volume and motility, especially sperm concentration and morphology in the oligozoospermia group, were significantly lower than in the normozoospermia group. The results of S-adenosylmethionine measurement showed that although there was a significant relationship between SAM levels in the 2 groups, these results clearly show a low S-adenosylmethionine level in the oligozoospermia group. The amount of SAM and its effects on sperm methylation and spermatogenesis can be effective. Complete spermatogenesis leads to correct sperm morphology and motility and finally increases the concentration of normal sperm. This was consistent with previous studies in which there was a significant relationship between spermatogenesis and the folate metabolism pathway, such as methylation (5). A study found that SAM induces hypermethylation in male infertility, and the methylation was significantly associated with male infertility (9). The results of the present study in the folate-related methylation pathway showed that most individuals in the oligozoospermia group had significantly lower MTHFR enzymatic activity than the normozoospermia group. These results were comparable to previous studies, and a study by Chan and colleagues in 2010 revealed that in mice with the *MTHFR* gene defection, low quality in both semen and sperm was observed. So there was consequently a defect in the spermatogenesis pattern and testicular tissue growth (21). The MTHFR enzyme is a key enzyme in the folate metabolism pathway, and by using NADPH causes an irreversible reaction that converts 5, 10-methylenetetrahydrofolate to 5-methyltetrahydrofolate (21).

SAM supplies the methyl group is in the cell, which is more important for DNA methylation and regulation of genes involved in spermatogenesis (13). Also, vitamin B12 is a critical coenzyme for the methylation of homocysteine to methionine with the conversion of 5-MTHF to THF (4, 11). Therefore, every change in enzymes involved in folate metabolism, such as MTHFR, may lead to male infertility (22, 23). The results of many studies showed that men with the *G1793AMTHFR* gene had an inverse relationship with sperm defects (24-28). Other studies found that infertile subjects receiving high doses of folic acid showed no improvement in the disease due to low levels of MTHFR enzyme protein and sperm DNA hypomethylation (29, 30). The results also showed that the MTHFR plays an important role in many diseases, including cardiovascular disease, nervous system diseases, especially central nervous system development in the fetal stage, diabetes, cancer, liver disease, and inflammatory diseases (31, 32). A similar study showed that the mutation MTHFR genotypes had a significant association with diseases such as pregnancy and infertility (33, 34). As noted in most studies, only the *MTHFR* gene has been investigated, so one of the innovations of the present study is to measure the activity of this enzyme by a manual method because the enzyme activity as a functional form can be more valuable for use in future diagnostic and therapeutic studies.

To conclude, there was a positive association between SAM levels and MTHFR activity in the 2 groups. It means that with increasing MTHFR activity, the amount of SAM increases. MTHFR activity and SAM levels similar to sperm parameters in the normozoospermia group were more than that seen in the oligozoospermia group. This indicates that MTHFR and its product SAM can be more effective in sperm development and complete spermatogenesis.

## 5. Conclusion

In the present study, it was observed that the level of SAM in the normozoospermia group was higher than in the oligozoospermia group. It was also found that the rate of MTHFR activity in normozoospermia individuals was significantly higher than oligozoospermia group. MTHFR activity and its product, SAM, play a role in sperm evolution, morphology, and motility. It seems that the activity of the MTHFR enzyme is an enzyme involved in folic acid metabolism and may be an important factor in spermatogenesis.

##  Conflicts of Interest

The authors declare no conflict of interest.
